# Evidence-based medicine and Management of Hepatocellular Carcinoma in Thalassemia

**DOI:** 10.1186/s12876-020-01542-2

**Published:** 2020-12-09

**Authors:** Andrea Mancuso

**Affiliations:** Medicina Interna 1, Azienda di Rilievo Nazionale ad Alta Specializzazione Civico - Di Cristina – Benfratelli, Piazzale Leotta 4, 90100 Palermo, Italy

**Keywords:** Thalassemia, Cirrhosis, Hepatocellular carcinoma, Liver transplant

## Abstract

**Background:**

Hepatocellular carcinoma as a complication is linked to improved outcomes of thalassemia.

**Main body:**

Published data suggest an incidence of HCC in thalassemia of about 2%. However, since thalassemia is endemic in many under-developed countries where patients have not probably been screened for HCC yet, the burden of the disease could be higher. Prevention of HCV infection through blood transfusion, HCV treatment and adequate iron chelation are all tools to prevent HCC in thalassemia. In presence of risk factors, HCC screening seems appropriate for thalassemia. Management of HCC should not be different from that indicated for non thalassemics. However, liver transplantation can be challenging and should be reserved to highly selected cases, due to coexistence of relevant comorbidities. Decisions in the management of HCC in thalassemia should follow a multidisciplinary effort. Moreover, due to the paucity of published data about the issue, future multicenter international studies will be helpful.

**Short conclusion:**

In BMC Gastroenterology results of a commendable effort to guidelines for the management of HCC in thalassemia are reported by an Italian panel of experts. However, due to the paucity of published data about the topic, some conclusions rely on grey areas and are reason of debate.

## Background

Thalassaemias are rare inherited haemoglobin disorders, endemic in the Mediterranean Area and South-East Asia. Depending on severity, there are two clinical forms: thalassemia major (TM), in which severe anemia starts during the first year of life, requires life-long periodic transfusion management; and thalassemia intermedia (TI), in which anemia has later onset and is generally milder, allowing survival without any regular transfusion [1]. However, today the TM-TI dichotomy should be considered outdated, and, for both clinical and research purposes, thalassemias should be divided in transfusion dependent (TDT) and non-transfusion dependent (NTDT).

## Main text

Many thalassaemics are infected with either hepatitis C or B virus, due to blood transfusions, particularly those who were born before the 1990s. Moreover, most of the patients have haemochromatosis, which is the main cause of morbidity and mortality [1]. In the last decades, a sort of revolution has been happening in the management of thalassemia. In fact, whether previously thalassemia was a fatal disease in the early adulthood because of iron overload related heart failure, the outcome has recently improved due to the introduction of iron chelating drugs. Consequently, hepatic disease, both due to iron overload and chronic hepatitis, in particular HCV, has recently become an important issue in thalassemia, being now the second cause of death after heart failure [2]. In this context, albeit somewhat obvious, the demonstration of efficacy of new oral antivirals for HCV treatment surely opens a window of hope and a new scenario [3]. In fact, the possibility of eradicating HCV, together with iron chelation, could potentially be able to reduce, if not to prevent, the burden of hepatic disease in thalassemia.

Until few decades ago, hepatocellular carcinoma (HCC) was not a relevant complication of thalassaemia, since heart failure affected survival and most cases died young. As a consequence of recent outcome improvement, due to iron chelation, both end-stage liver disease and HCC have become a relevant issue [1, 4–6].

A multicenter retrospective study identified 22 cases of HCC in Italian patients with thalassemia syndromes, 19 of whom with either TDT or NTDT [7]. Subsequently, single cases of HCC were described in thalassemia without cirrhosis [8]. Finally, 2% HCC incidence in thalassemia was reported by a pivotal prospective study [9]. Moreover, a recent retrospective study reported 62 new cases among 5855 thalassaemia patients [10].

In BMC Gastroenterology, a panel of authors from different areas of Italy, report the result of a commendable effort to guidelines for the management of HCC in thalassemia, suggesting some detailed indication on most of the main issues about the topic [11].

As an external observator, I would firstly wonder whether the time is mature for agreeable guidelines. However, the answer is not obvious because time is surely mature for discussion of this hot topic, but, as often for rare diseases, evidence is scarse and could hardly be the only support of absolute indications by any guidelines. Consequently, in absence of solid evidence, experts opinion and compromise through discussion could lead indication in guidelines.

Specifically, I will concentrate on some of the main debated issues about management of HCC in thalassemia.

In fact, the difference of HCC incidence in patients with and without thalassemia is a question without a sure answer, since thalassemia is rare and HCC started to develop as a consequence of improved thalassemia outcome. Certainly, HCC seems to arise in thalassemia at a younger age than in non thalassemics [9, 10], but further data are needed to provide confidence.

Furthermore, in the context of thalassemia, the importance of screening for HCC with respect to risk factors, chronic iron accumulation alone or in combination with long standing hepatitis C even after eradication with the new DAAs should be emphasized.

Another debated topic is whether HCC is prevalent in non cirrhotic thalassemics or in NTDT than in TDT. In fact, a significant rate of HCC in thalassemia is reported in non cirrhotic liver and mainly in NTDT [7, 9, 10]. However, two possible bias can affect this observation. The former is that, being patients with thalassemia not followed up in a hepatological department and rarely undergoing liver biopsy, a significant rate of cases could be misdiagnosed, having compensated cirrhosis. The latter is that better outcome of NTDT could have allowed HCC development in NTDT more than in TDT [1]. Moreover, HCC is likely under-reported in thalassemia today and management possibly inaccurate since experts in thalassemia are generally not skilled in hepatology and vice versa [12].

A further controversial issue is about recall policy of HCC. In fact, whether several centers propose that it should be based on ultrasound and alphafetoprotein every 6 months and MRI according to liver iron concentration (LIC), it has very little support by published data. In fact, in real life, other groups practically follow the same recall policy as in non thalassemics [4].

Finally, there is a debate about treatment of HCC. In fact, with the exception of liver transplantation (LT), treatment of HCC should simply follow the same indication as in non thalassemics. Moreover, due to the outcome dramatic improvement, it is now time to dispel the myth that thalassemia is a contraindication to HCC treatments that are effective in non-thalassemia patients [1, 2, 4, 6].

Some more comment is needed about the option of LT in thalassemia, a controversial issue yet. Furthermore, most still consider LT a contraindication in thalassemia, because of both lack of published evidence and little confidence with thalassemia management. However, due to recent improvement in the outcome of thalassemia, a reconsideration of the issue would be appropriate. In fact, once significant comorbidities are excluded, namely severe pulmonary hypertension and overt heart disease, LT seems an acceptable option for selected cases [4, 13–19]. However, published data about combined heart and liver transplantation in thalassemia are insufficient for any conclusion [20].

## Conclusions

In conclusion, as for other rare diseases, decisions on the management of HCC in thalassemia are not based on strong evidence. Consequently, experience in the field is the main rule that can address decisions and collegial multidisciplinary management is the milestone.

## Data Availability

Not applicable.

## Abbreviations

TM: Thalassemia major
TI: Thalassemia intermedia
HCC: Hepatocellular carcinoma
LT: Liver transplantation

## References

[1] Motta I, Mancarella M, Marcon A, Vicenzi M, Cappellini MD (2020). Management of age-associated medical complications in patients with β-thalassemia. Expert Rev Hematol.

[2] Moukhadder HM, Halawi R, Cappellini MD, Taher AT (2017). Hepatocellular carcinoma as an emerging morbidity in the thalassemia syndromes: a comprehensive review. Cancer.

[3] Mangia A, Sarli R, Gamberini R, Piga A, Cenderello G, Piazzolla V, Santoro R, Caruso V, Quarta A, Ganga R, Copetti M, Forni G (2017). Randomised clinical trial: sofosbuvir and ledipasvir in patients with transfusion-dependent thalassaemia and HCV genotype 1 or 4 infection. Aliment Pharmacol Ther.

[4] Mancuso A (2010). Hepatocellular carcinoma in thalassemia: a critical review. World J Hepatol.

[5] Mela M, Mancuso A, Burroughs AK (2003). Hepatocellular carcinoma: indications for liver transplantation. Aliment Pharmacol Ther.

[6] Mancuso A (2013). Management of hepatocellular carcinoma: enlightening the gray zones. World J Hepatol.

[7] Borgna-Pignatti C, Vergine G, Lombardo T, Cappellini MD, Cianciulli P, Maggio A, Renda D, Lai ME, Mandas A, Forni G, Piga A, Bisconte MG (2004). Hepatocellular carcinoma in the thalassaemia syndromes. Br J Haematol.

[8] Mancuso A, Rigano P, Renda D, Di Salvo V, Pignatti CB, Guddo F, Buccellato A, Nicoli N, Maggio A (2005). Hepatocellular carcinoma on cirrhosis-free liver in a HCV-infected thalassemic. Am J Hematol.

[9] Mancuso A, Sciarrino E, Renda MC, Maggio A (2006). A prospective study of hepatocellular carcinoma incidence in thalassemia. Hemoglobin.

[10] Restivo Pantalone G, Renda D, Valenza F, D’Amato F, Vitrano A, Cassarà F, Rigano P, Di Salvo V, Giangreco A, Bevacqua E, Maggio A (2010). Hepatocellular carcinoma in patients with Thalassaemia Syndromes: clinical characteristics and outcome in a long term single Centre experience. Br J Haematol.

[11] Mangia A, Bellini D, Cillo U (2020). Hepatocellular carcinoma in adult thalassemia patients: an expert opinion based on current evidence. BMC Gastroenterol.

[12] Mancuso A (2017). Management of hepatocellular carcinoma in thalassemia and importance of the human factor. Cancer.

[13] Mancuso A (2014). Hepatocellular carcinoma in thalassemia: emerging issues and challenges for liver transplant. Aliment Pharmacol Ther.

[14] Mancuso A, Perricone G (2015). Time to define a new strategy for management of hepatocellular carcinoma in thalassaemia?. Br J Haematol.

[15] Mancuso A, Perricone G (2014). Hepatocellular carcinoma and liver transplantation: state-of-the-art. J Clin Transl Hepatol.

[16] Cartin-Ceba R, Krowka MJ (2019). Pulmonary complications of portal hypertension. Clin Liver Dis.

[17] Cohen AR, Galanello R, Pennell DJ, Cunningham MJ, Vichinsky E (2004). Thalassemia. Hematol Am Soc Hematol Educ Program..

[18] Mancuso L, Scordato F, Pieri M, Valerio E, Mancuso A (2013). Management of portopulmonary hypertension: new perspectives. World J Gastroenterol.

[19] Maffei L, Sorrentino F, Caprari P, Taliani G, Massimi S, Risoluti R, Infection in Thalassemia Syndromes MSHCV (2020). Hemoglobinopathies: new perspectives. Front Mol Biosci.

[20] Olivieri NF, Liu PP, Sher GD, Daly PA, Greig PD, McCusker PJ, Collins AF, WH Francombe DM, Butany J (1994). Combined liver and heart transplantation for end-stage Iron-induced organ failure in an adult with homozygous Beta-Thalassemia. N Engl J Med.

